# Ultrastructure and Dynamics of Synaptonemal Complex Components During Meiotic Pairing and Synapsis of Standard (A) and Accessory (B) Rye Chromosomes

**DOI:** 10.3389/fpls.2019.00773

**Published:** 2019-06-20

**Authors:** Susann Hesse, Mateusz Zelkowski, Elena I. Mikhailova, Christian J. Keijzer, Andreas Houben, Veit Schubert

**Affiliations:** ^1^Leibniz Institute of Plant Genetics and Crop Plant Research (IPK) Gatersleben, Seeland, Germany; ^2^N.I.Vavilov Institute of General Genetics, Russian Academy of Sciences, Saint-Petersburg State University, Saint-Petersburg, Russia; ^3^Innovert GBVM, Vlierden, Netherlands

**Keywords:** B chromosomes, CENH3, meiosis, recombination, *Secale cereale*, scanning electron microscopy, super-resolution microscopy, synaptonemal complex

## Abstract

During prophase I a meiosis-specific proteinaceous tripartite structure, the synaptonemal complex (SC), forms a scaffold to connect homologous chromosomes along their lengths. This process, called synapsis, is required in most organisms to promote recombination between homologs facilitating genetic variability and correct chromosome segregations during anaphase I. Recent studies in various organisms ranging from yeast to mammals identified several proteins involved in SC formation. However, the process of SC disassembly remains largely enigmatic. In this study we determined the structural changes during SC formation and disassembly in rye meiocytes containing accessory (B) chromosomes. The use of electron and super-resolution microscopy (3D-SIM) combined with immunohistochemistry and FISH allowed us to monitor the structural changes during prophase I. Visualization of the proteins ASY1, ZYP1, NSE4A, and HEI10 revealed an extensive SC remodeling during prophase I. The ultrastructural investigations of the dynamics of these four proteins showed that the SC disassembly is accompanied by the retraction of the lateral and axial elements from the central region of the SC. In addition, SC fragmentation and the formation of ball-like SC structures occur at late diakinesis. Moreover, we show that the SC composition of rye B chromosomes does not differ from that of the standard (A) chromosome complement. Our ultrastructural investigations indicate that the dynamic behavior of the studied proteins is involved in SC formation and synapsis. In addition, they fulfill also functions during desynapsis and chromosome condensation to realize proper recombination and homolog separation. We propose a model for the homologous chromosome behavior during prophase I based on the observed dynamics of ASY1, ZYP1, NSE4A, and HEI10.

## Introduction

Meiosis is a type of cell division that reduces the chromosome number by half, and creates haploid cells. This type of cell division is a fundamental and evolutionary conserved process in all sexually reproducing eukaryotes and is characterized by four main chromosomal processes. First, sister chromatid cohesion becomes established during S phase by cohesin complexes. Second, the chromosome axis condenses and pairing of homologous chromosomes takes place. Third, the synaptonemal complex (SC) is formed via synapsis and fourth, recombination occurs eventually leading to crossover formation (Sanchez-Moran et al., 2008). In addition, homology-dependent or -independent interactions, e.g., centromere and/or telomere clustering can prelude and/or complement these processes (Zickler and Kleckner, 2015). The segregation of homologous chromosomes to the opposite poles of the spindle during meiosis I is followed by the second part of meiosis (meiosis II), which leads to the formation of four daughter cells. In species with monocentric chromosomes meiosis II resembles a mitotic division in terms of sister chromatid separation.

Only few organisms exhibit a deviating program of prophase I events. In most species SC formation depends on double strand break (DSB) formation and strand invasion. However, in e.g., *Caenorhabditis elegans* and *Drosophila* females SC formation occurs without DSB formation (Zickler and Kleckner, 2015). In *Schizosaccharomyces pombe* and *Aspergillus nidulans* no SCs become established and pairing occurs recombination-independent and recombination-mediated, respectively (Olson et al., 1978; Egel-Mitani et al., 1982; Bahler et al., 1993).

Studies across yeast, mammals and plants indicate that the SC structure is as highly conserved as meiosis itself (Zickler and Kleckner, 1999, 2015; Page and Hawley, 2004). Early transmission electron microscopy revealed the basic SC organization as a tripartite structure consisting of two lateral elements (LEs) flanking a ~100 nm wide central region (CR) (Fawcett, 1956; Moses, 1956, 1968). Prior to SC formation, axial element (AE) components assemble alongside the cohesin-based chromosome axis mediating sister chromatid cohesion, to establish the meiotic chromatin loop-axis structure (Zickler and Kleckner, 1999). During synapsis, homologous AEs are linked in a zipper-like manner by CR components along their entire length. With a diameter of about 50 nm, the AEs are called lateral elements (LEs) within the SC (Moses, 1968; Westergaard and von Wettstein, 1972). The CR consists of two functional units, namely the transverse filament (TF) proteins that span the CR to link both homologous chromosomes, as well as central region proteins acting tentatively to stabilize the CR (De Vries et al., 2005; Bolcun-Filas et al., 2007, 2009; Hamer et al., 2008; Page et al., 2008; Schramm et al., 2011; Humphryes et al., 2013; Collins et al., 2014; Hernandez-Hernandez et al., 2016).

Genomic and proteomic approaches, e.g., in budding yeast, identified multiple genes and proteins involved in SC formation and meiotic processes that appear to have orthologs across various eukaryotes (Zickler and Kleckner, 1999, 2015; Page and Hawley, 2004; Gerton and Hawley, 2005). Despite the common basic structural similarity between SCs, primary amino acid sequence comparisons of orthologs components show a substantial dissimilarity. For example the TF protein ZYP1 of *Arabidopsis thaliana* (L.) Heynh shares only 18–20% sequence identity and 36–40% similarity with the corresponding proteins of budding yeast (ZIP1), *Drosophila* (C(3)G) and rat (SCP1) (Meuwissen et al., 1992; Sym et al., 1993; Page and Hawley, 2001; Higgins et al., 2005). Furthermore, orthologous genes do not necessarily encode proteins with equivalent functions. For instance, electron microscopy confirmed that the ASY1 protein of *A. thaliana* belongs to the axis-associated proteins, whereas its ortholog of budding yeast (HOP1) is crucial for AE formation (Hollingsworth and Ponte, 1997; Armstrong et al., 2002). In summary, the studies of SC components suggest that their evolution was driven by the need to fulfill a structural role, rather than conserving a catalytic one (Zickler and Kleckner, 2015).

Beside ASY1 and ZYP1, additional components, such as subunits of the structural maintenance of chromosome (SMC)5/6 complex and human enhancer of invasion-10 (HEI10) proteins associated with the chromosome axis (axial/lateral element) have been identified. Components of the plant chromosome axis comprise in addition HORMA domain containing proteins (Armstrong et al., 2002; Nonomura et al., 2006), coiled-coil proteins (Wang et al., 2011; Ferdous et al., 2012; Lee et al., 2015) and cohesins (Cai et al., 2003; Lam et al., 2005).

The conserved SMC5/6 complex belongs to the SMC family which is formed via the interaction of the hinge domains of the SMC5 and SMC6 subunits resulting in a heterodimer connected by the δ-kleisin NSE4 (NON-SMC ELEMENT 4) at the head domains of SMC5 and SMC6 (Lehmann et al., 1995; Fousteri and Lehmann, 2000; Palecek et al., 2006; Taylor et al., 2008). In addition to functions of SMC5/6 in somatic tissues, various essential roles during meiosis were found in yeasts, worm, mouse and human. SMC5/6 subunits were proven to play a role in meiotic processes such as in response to double strand breaks (DSBs), meiotic recombination, heterochromatin maintenance, centromere cohesion, homologous chromosome synapsis and meiotic sex chromosome inactivation (Verver et al., 2016). In *A. thaliana*, due to the presence of two alternative SMC6 (SMC6A and SMC6B) and NSE4 (NSE4A and NSE4B) subunits, different SMC5/6 complexes may be composed (Schubert, 2009; Zelkowski et al., 2019).

HEI10 is a member of the ZMM (ZIP1/ZIP2/ZIP3/ZIP4, MSH4/MSH5, and MER3) protein family, originally identified as a growth regulator and essential for meiotic recombination in different eukaryotes (Toby et al., 2003; Whitby, 2005; Osman et al., 2011; Chelysheva et al., 2012; Wang et al., 2012). Possessing a RING-finger motif, coiled-coil and tail domains, HEI10 functions as an E3 ligase catalyzing post-translational protein modification by ubiquitin-like proteins and thereby integrates different meiotic processes for successful recombination (De Muyt et al., 2014; Qiao et al., 2014).

In the past, in plants such as *A. thaliana* as well as large-genome cereals important meiotic studies were performed. The cereal species rye (*Secale cereale* L.) contains, in addition to the standard A chromosome (As) complement, dispensable accessory chromosomes, also called B chromosomes (Bs). The number of Bs varies between individuals of a population. Bs were reported in thousands of eukaryotic species, but so far remain an evolutionary mystery. Apart from other peculiarities, Bs do not pair or recombine with As at meiosis and often exhibit a non-Mendelian inheritance (Houben et al., 2014). Previous studies by electron microscopy showed that the synaptic behavior of rye Bs differs from that of As. In addition to bivalent formation, Bs may also perform intrachromosomal synapsis and form multivalents (Santos et al., 1993, 1995; Jiménez et al., 1994). However, the SC protein composition of As and Bs has not yet been investigated in detail.

Despite extensive studies on the assembly of SCs, much less is known about the process of SC disassembly, which is essential for correct chromosome segregation (Cahoon and Hawley, 2016). In this study, we used super-resolution and electron microscopy to monitor the dynamics of ultrastructural changes during the assembly and disassembly of SCs in rye plants containing Bs at a resolution beyond widefield microscopy. Immunohistochemistry allowed us to track the four meiotic proteins ASY1 (a marker for AE/LE), ZYP1 (a transverse filament protein), HEI10 (a structure-based signal transduction protein involved in recombination), and NSE4A (a δ-kleisin of the SMC5/6 complex) during prophase I. Until the complete disassembly, all four proteins were present at the SC. Their spatio-temporal distribution revealed extensive chromatin structure changes.

## Materials and Methods

### Plant Material

Rye (*Secale cereale* L. cv. Paldang) plants carrying B chromosomes (2*n* = 14+0−4 supernumerary Bs) (Romera et al., 1989) were grown under greenhouse conditions (22°C, 16 h light/8 h dark) to obtain anthers containing pollen mother cells (PMCs) during prophase I. The number of Bs in individual plants was determined by FISH using rye B chromosome-specific probes.

### FISH Probe Preparation

The retrotransposon Bilby (Francki, 2001) was used as centromere-specific probe, and the repeats Sc11, Sc55c1, Sc63c34, D1100, E3900, and Sc36c82 were employed as rye B chromosome-specific probes (Sandery et al., 1990; Blunden et al., 1993; Klemme et al., 2013). Labeling was done by nick translation using a NT Labeling Kit (Jena Bioscience GmbH, Jena Germany).

### Identification of B Chromosome Number

Root tips of each rye plant were cut and in fixed in ethanol/acetic acid (3:1) for 48 h at room temperature. The fixed roots were stained in 1% acetocarmine solution (1% carmine in 45% acetic acid, 12–24 h at room temperature). For slide preparation the roots were carefully heated up in the acetocarmine solution over an open flame until they became soft. Then, the soft roots were placed on a slide, the root tip cap was cut off with a razor blade and the meristem was carefully extracted on the slide by use of a preparation needle. The extracted meristem was squashed in 45% acetic acid using a coverslip. After coverslip removal using liquid nitrogen, the slides were stored in 100% ethanol (4°C). Subsequently, the slides were air-dried and the FISH probe-containing hybridization mix (FISH probes diluted in 20% dextran sulfate, Sigma-Aldrich, cat. no. D 8906, 50% deionized formamide, 300 mM NaCl, 30 mM tri-sodium citrate dehydrate, 50 mM phosphate buffer, pH 7.0) was applied. Then, the slides were incubated for denaturation for 2 min at 80°C in darkness. FISH was performed at 37°C overnight. Slides were washed 3 ×5 min in 1 × PBS and afterwards mounted and counterstained with 4′,6-diamidine-2′-phenylindole dihydrochloride (DAPI, 1 mg/ml) in Vectashield (Vector Laboratories). To determine the number and type of B chromosomes (Endo et al., 2008), FISH probes directed against the pericentromeric repeat Sc11 and a subtelomeric repeat (E3900 or D1100) were used in parallel. In case of standard rye B chromosomes the detected number of both repeats is equal. Plants containing standard Bs were cultivated further under greenhouse conditions (22°C, 16 h light/8 h dark) for this study.

### Immunostaining and FISH on Meiotic Chromosomes

Rye anthers with meiocytes at prophase I were fixed 25 min under vacuum in 4% ice-cold paraformaldehyde in 1 × PBS (phosphate buffer saline, pH 7.4), washed 3 ×5 min in ice-cold 1 × PBS and 20 min digested at 37°C in an enzyme cocktail (0.1% cellulose, Calbiochem, cat. no. 219466; 0.1% pectolyase Y-23, Sigma-Aldrich, cat. no. P3026; 0.1% cytohelicase, Sigma-Aldrich, cat. no. C8274) in 1 × PBS. After washing 3 ×5 min in ice-cold 1 × PBS, single anthers were transferred to slides and squashed in 1 × PBS+0.001% Tween-20 using coverslips. After coverslip removal using liquid nitrogen, the slides were stored in 1 × PBS. For longer storage they were transferred to 100% glycerol (Carl Roth, cat. no. 3783) and kept at 4°C. The following primary antibodies were applied at 37°C for 90 min: rabbit anti-*Zea mays* ASY1 (1:200), guinea pig anti-*Zea mays* ZYP1 (1:200; Golubovskaya et al., 2011), rabbit anti-*A. thaliana* NSE4A (1:200; Zelkowski et al., 2019), mouse anti-*Oryza sativa* HEI10 (1:200; Wang et al., 2012), and rabbit anti-grass CENH3 (1:1,000; Sanei et al., 2011). For detection, the following secondary antibodies were applied at 37°C for 60 min: goat anti-rabbit Dylight488 (1:200; Dianova cat. no. 111-485-144), goat anti-guinea pig Alexa Fluor594 (1:400; Molecular Probes cat. no. A11076), goat anti-mouse Cy3 (1:400; Dianova cat. no. 115-166-146), and donkey anti-guinea pig Alexa Fluor647 (1:200; Dianova cat. no. 706-605-148). Afterwards, the slides were washed in 3 ×5 min 1 × PBS, dehydrated (2 min each step; 70, 90, and 100% ethanol), air-dried and fixed in ethanol/acetic acid (3:1; 24–48 h in darkness at room temperature). Subsequently, the slides were air-dried and incubated with the FISH probe-free hybridization mix (see above) for 12 h at 37°C. After short washing for 5 min in 2 × SSC containing 0.1% Triton X100, the slides were dehydrated and air-dried. Then, for DNA denaturation, slides were incubated in 0.2 N NaOH (in 70% ethanol; 10 min at room temperature), dehydrated and air-dried. Subsequently, the FISH probes were diluted and denatured for 5 min at 95°C in the hybridization mix before application on slides. FISH was performed at 37°C overnight using Bilby or the B-specific probes. Slides were washed 3 ×5 min in 1 × PBS and afterwards mounted and counterstained with DAPI, (1 mg/ml) in Vectashield (Vector Laboratories).

### Determination of Meiotic B Chromosome Pairing Configurations

To determine the meiotic pairing behavior of rye B chromosomes, immunostaining using the primary antibodies directed against *Zea mays* ASY1 and *Z. mays* ZYP1, and subsequent FISH using a cocktail of the rye B chromosome-specific probes Sc11, Sc55c1, Sc63c34, D1100, E3900, and Sc36c82 was performed on meiocytes as described above. The determination of pairing configurations was done using a BX61 microscope (Olympus) equipped with an ORCA-CCD camera (Hamamatsu) or by super-resolution microscopy. For quantification only meiocytes with completed synapsis were considered.

### Super-Resolution Microscopy

To analyse the ultrastructure of immunosignals and chromatin beyond the classical Abbe/Raleigh limit at a lateral resolution of ~120 nm (super-resolution, achieved with a 488 nm laser) spatial structured illumination microscopy (3D-SIM) was applied using a 63 × /1.4 Oil Plan-Apochromat objective of an Elyra PS.1 microscope system and the software ZENblack (Carl Zeiss GmbH). Images were captured separately for each fluorochrome using the 642, 561, 488, and 405 nm laser lines for excitation and appropriate emission filters (Weisshart et al., 2016). Maximum intensity projections of whole meiocytes were calculated via the ZEN software. Zoom in sections were presented as single slices to indicate the subnuclear chromatin and protein structures at the super-resolution level. 3D rendering and CENH3 volume measurements based on SIM image stacks was done using the Imaris 8.0 (Bitplane) software.

### Scanning Electron Microscopy

Anthers of *S. cereale* were cut into equal halves. In order to determine the meiotic stage, one half was fixed in ethanol-acetic acid (3:1). Then, spread preparations of the fixed anthers containing PMCs at different meiotic stages were made according to Zhong et al. (1996). The preparations were air-dried, mounted in DAPI-Vectashield and observed using fluorescence microscopy. Alternatively, they were fixed in ethanol-acetic acid (3:1), stained with acetocarmine and observed with bright field microscopy. The complementary half was fixed in 70% ethanol, frozen by plunging into liquid propane at −180°C, cryo-fractured using a nitrogen-cooled razor blade and thawed to room temperature in 70% ethanol. This complementary half was dehydrated in 100% ethanol and critical point dried over carbon dioxide. Subsequently, it was mounted on a stub with the fractured plane up, coated with 2 nm platinum and observed in a JEOL 6300F field emission scanning electron microscope (SEM) at 5 kV.

After extensive trials looking for the best fixative for this purpose, 70% ethanol proved to produce the best images in SEM compared to the more advanced fixatives as glutaraldehyde and osmium tetroxide which are generally used for observing phospholipid- and protein-related structures in electron microscopy.

## Results

**Figure 1:**
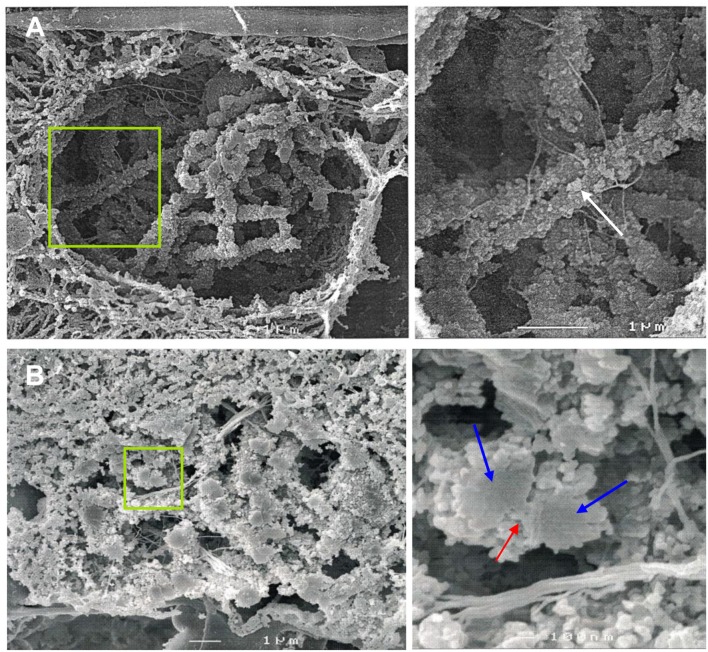
SEM imaging reveals the ultrastructure of rye bivalents during prophase I. The images of the right column show the regions of interest enlarged. **(A)** Top view of aligned homologous chromosomes inside a meiocyte at zygotene. Chromatin clusters (chromomeres) are clearly visible at the chromosome surface (arrow). **(B)** Cross section of a bivalent inside a meiocyte during zygotene-pachytene. The bivalent in the green rectangle is composed of two paired homologs (blue arrows) both containing two chromatids. The red arrow indicates the SC.

**Figure 2:**
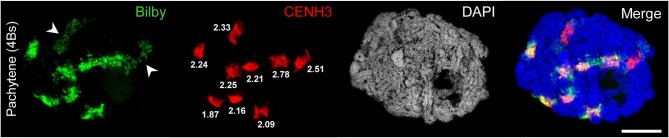
Bilby repeats and CENH3 identify the centromeres of A and B chromosomes. A rye meiocyte containing four Bs at pachytene shows the pairing of all centromeres labeled with Bilby and CENH3 at the centromeric regions. The brighter Bilby signals reflect the seven (peri)centromeric regions of the A bivalents. In contrast, the Bilby signals of the Bs (arrowheads) appear darker and less condensed. Interestingly, this difference is not revealed by means of the CENH3 labeling implying that the actual size of active centromeres does not differ between A and B chromosomes. The similar CENH3 volumes (μm^3^) are indicated at the signals. The global chromatin staining with DAPI dicerns the chromatin-free SC structures. Bar = 5 μm.

**Figure 3:**
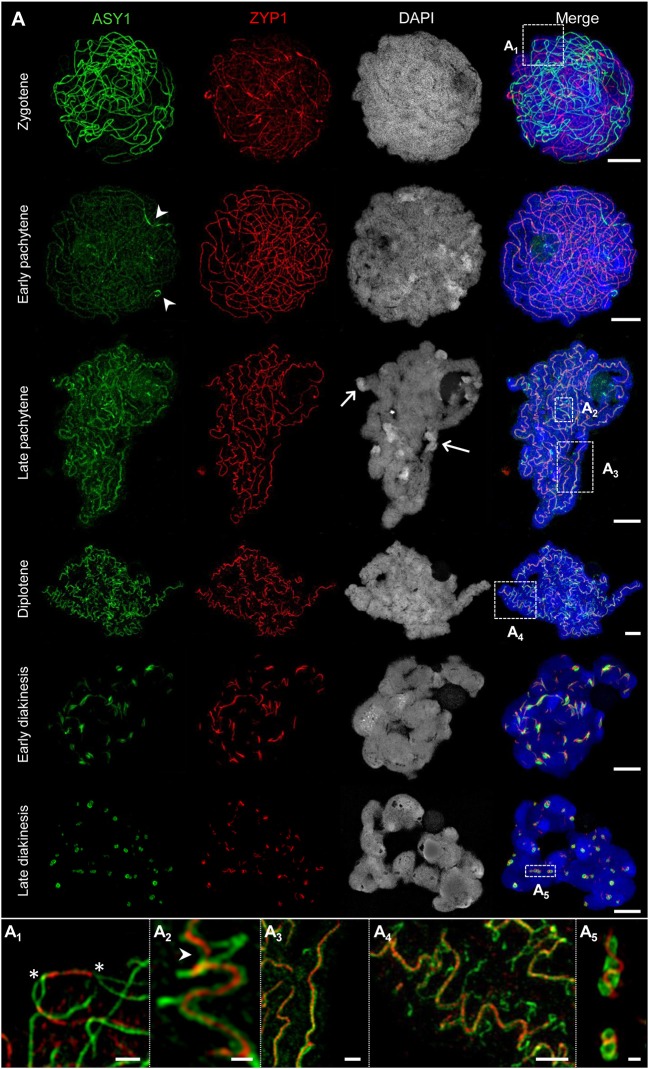
The behavior of ASY1 and ZYP1 during prophase I. The images **A**_**1**_**-A**_**5**_ show enlarged regions delimited by dashed boxes. Chromatin was stained with DAPI. **(A)** Representative examples of immunostaining of ASY1, a marker for chromosome axis, and ZYP1, a SC transverse filament protein. At zygotene, intense ASY1 signals are visible along not yet synapsed chromatin axes. When synapsis proceeds in early pachytene, the SCs assemble at multiple sites of the chromatin and the ZYP1 signals become more prominent. The ASY1 signal intensity strongly decreases at synapsed regions, but never vanishes completely. At early pachytene, all homologs are synapsed. The last separated regions can be identified by brighter ASY1 signals (arrowheads). At late pachytene, the ongoing chromatin condensation causes a twisted SC structure. ASY1 starts separating from ZYP1, reflecting the initiation of SC disintegration **(A**_**2**_**,A**_**3**_**)**. Note the regions with a substantially higher DAPI staining intensity at the telomeric heterochromatin (arrows) corresponding to increased chromatin condensation. At diplotene, the SCs form spiral-like structures with ASY1 strands retracting from the SC at multiple positions **(A**_**4**_**)**, which reflects proceeding SC disassembly and further chromatin condensation. At early diakinesis, ZYP1 staining detects only short SC fragments enwrapped by ASY1. At late diakinesis, only compact ball-like ASY1 structures with embedded ZYP1 remain **(A**_**5**_**)**, Figure 5E; Supplementary Movie 2). They disappear completely until the end of diakinesis (Figure 6E). Bars = 5 μm. **(A**_**1**_**)** An interstitial synapsis initiation site showing ZYP1 signals flanked by still separated ASY1 strands (asterisks, see also Supplementary Movie 3). Bar = 1 μm. **(A**_**2**_**)** Initiation of SC disintegration at late pachytene. ASY1 strands dissociate from single SC sites via loop formation. At positions where both ASY1 strands become retracted from the SC, ZYP1 disappears (arrowhead). Bar = 0.5 μm. **(A**_**3**_**)** ZYP1 enwinded in two ASY1 strands during late pachytene. Bar = 1 μm. **(A**_**4**_**)** At diplotene, the ASY1 structures dissociate from the SC and start to dissolve at various positions indicating multiple desynapsis sites. Bar = 2 μm. **(A**_**5**_**)** At late diakinesis, short ZYP1 fragments are embedded in ball-like ASY1 structures. Bar = 0.5 μm.

**Figure 4:**
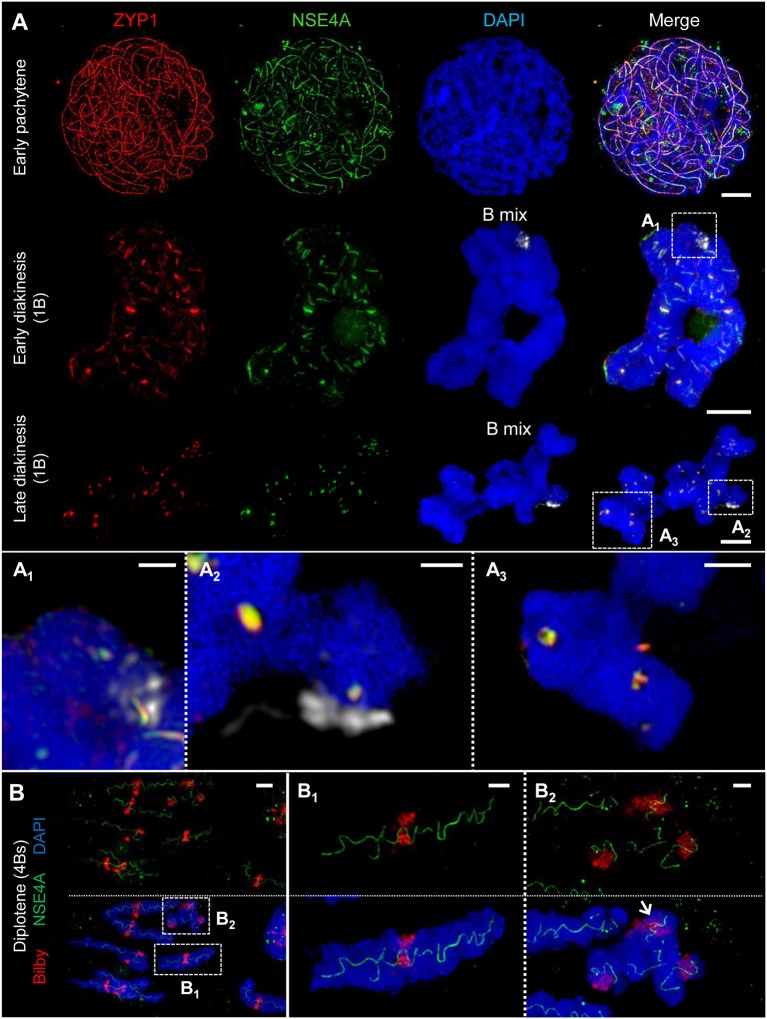
ZYP1 and NSE4A colocalize at the SCs of rye A and B chromosomes. The images **(A**_**1**_**-A**_**3**_**,B**_**1**_**,B**_**2**_**)** show enlarged regions delimited by dashed boxes. The B chromosomes were detected by a B-specific FISH probe mix, centromeres by the specific repeat Bilby. The global chromatin was stained with DAPI. **(A)** Simultaneous immunolocalization of NSE4A and ZYP1 shows clearly their co-localization at the central region of the SC throughout prophase I. At pachytene, the NSE4A signals are present along the synapsed homologs. When degradation proceeds during diakinesis, NSE4A can be detected only at the ZYP1-positive SC fragments. A and B chromosomes behave similar **(A**_**1**_**-A**_**3**_**)**. Bars = 5 μm. **(A**_**1**_**)** A self-pairing rye B chromosome manifests the co-localization of NSA4A and ZYP1 at early diakinesis. Bar = 1 μm. **(A**_**2**_**)** At late diakinesis, a self-paired rye B chromosome displays typical ball-like residual structures of the SC complex identical to those present on A chromosomes. NSE4A and ZYP1 colocalize. Bar = 2 μm. **(A**_**3**_**)** An A chromosome bivalent showing ball-like structures of the remaining SC at late diakinesis. Note the colocalisation of NSE4A and ZYP1. Bar = 2 μm. **(B)** A meiocyte with seven A homologs and four B chromosomes (4Bs) at diplotene. The twisted NSE4A signals follow the SC structure typically present at diplotene. Bilby identifies the centromeres of the bivalents. The NSE4A structures are identical at A and B chromosomes **(B**_**1**_**,B**_**2**_**)**. Bars = 5 μm. **(B**_**1**_**)** Typical twisted NSA4A structure of an A bivalent. Bar = 2 μm. **(B**_**2**_**)** Twisted NSE4A structures indicate the SCs at four B chromosomes forming a multivalent. The Bs can be distinguished from As by their smaller size and the increased Bilby signal dispersion. Two of the B centromeres are associated (arrow). Bar = 2 μm.

**Figure 5:**
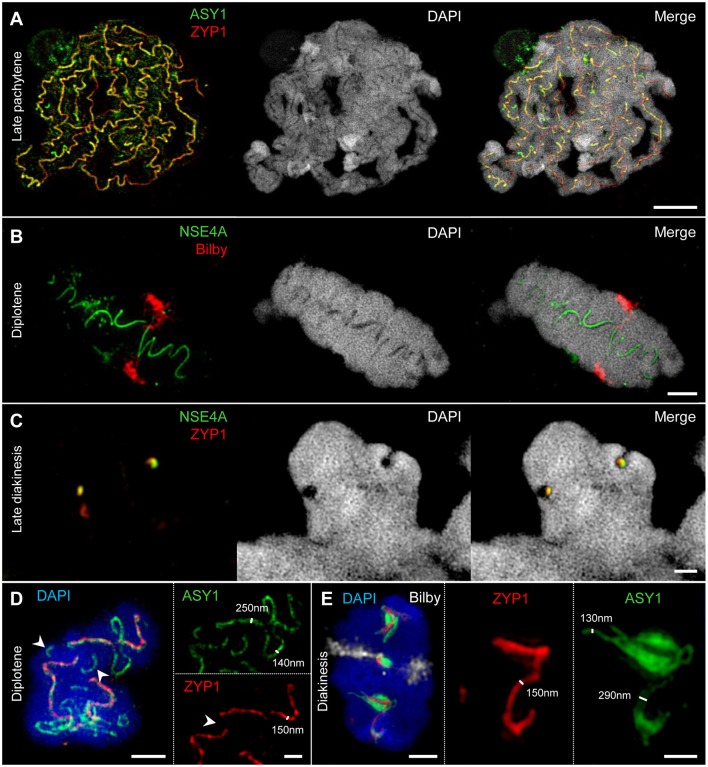
SIM identifies the SC as a complex protein structure embedded in chromatin. Chromatin was stained with DAPI. **(A–C)** At different stages of prophase I, chromatin-free structures can be visualized within paired homologs. They comprise co-localized ASY1, ZYP1, and NSE4A proteins indicating the inner SC as chromatin-free. Bars = 5 μm **(A)**, 2 μm **(B)**, 1 μm **(C)**. **(D)** An A chromosome bivalent at diplotene showing SC disintegration accompanied by the retraction of ASY1 from the SC. While ASY1 forms loop structures at early desynapsis, ZYP1 disappears at positions were synapsis is already resolved (arrowheads). Bars = 2 μm (bivalent), 1 μm (enlarged region). **(E)** At diakinesis ASY1 winds up around the short residual ZYP1 strands at few positions suggesting a special role of these emerging ball-like structures. The centromeres were labeled with Bilby (see also Supplementary Movie 2). Bars = 2 μm (bivalent), 1 μm (enlarged region). The SIM resolution allows to measure the relative width of the ASY1 and ZYP1 structures at different prophase I stages. Single ASY1 strand have about the same width as ZYP1 strands **(D,E)**.

**Figure 6:**
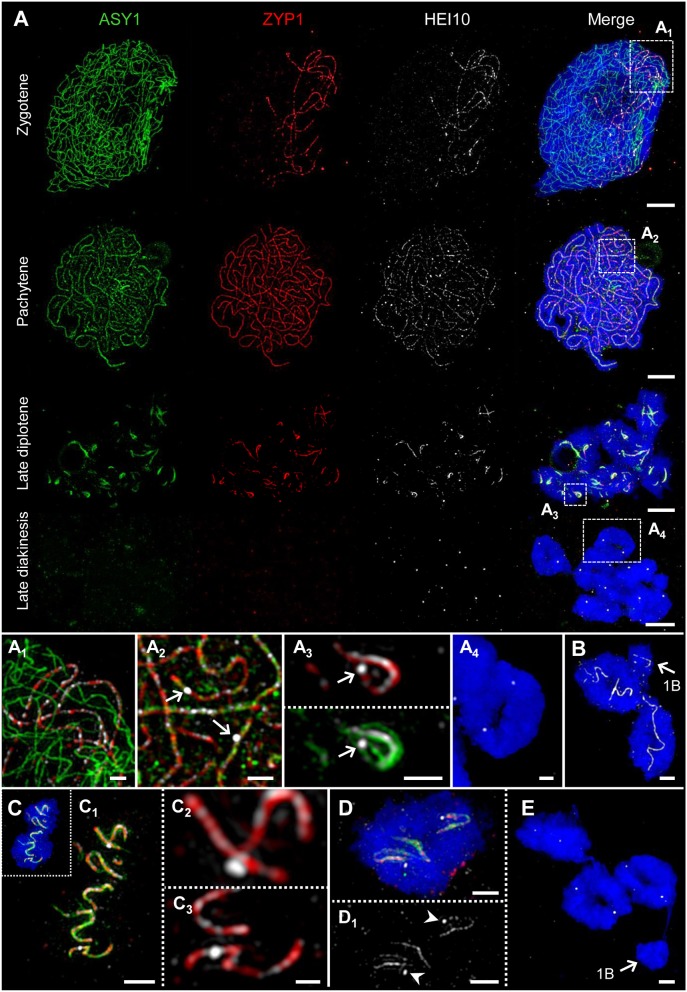
HEI10 behavior compared to ASY1 and ZYP1 dynamics during prophase I. The images **(A**_**1**_**-A**_**4**_**)** show enlarged regions delimited by dashed boxes. Chromatin was counterstained with DAPI. **(A)** Throughout prophase I until late diplotene, HEI10 foci follow the dynamics of ZYP1. At zygotene, HEI10 foci are present at the central region of the SC marked by ZYP1. At the end of synapsis during pachytene single HEI10 foci become more prominent. At diplotene, the progression of SC disassembly causes the fragmentation of the SC, and HEI10 can be detected either as numerous low-intensity foci organized along the central element, or as a few prominent foci most likely corresponding to crossover- fated recombination sites. At late diakinesis, ASY1 and ZYP1 disappear, but HEI10 proteins remain as distinct spots at the potential recombination sites. Bars = 5 μm. **(A**_**1**_**)** At zygotene, HEI10 foci occur exclusively in SCs marked by ZYP1. Bar = 1 μm. **(A**_**2**_**)** At pachytene, individual HEI10 foci become more pronounced and clearly distinguishable (arrows). Bar = 1 μm. **(A**_**3**_**)** Low-intensity HEI10 foci along the residual central region of the SC exist in parallel to a pronounced HEI10 focus (arrow) indicating a recombination site at late diplotene. ASY1 threads coil up at this position. Bar = 1 μm. **(A**_**4**_**)** An A chromosome ring bivalent at late diakinesis with two HEI10 spots marking the potential sites of crossovers. ASY1 and ZYP1 signals are no longer detectable. Bar = 1 μm. **(B)** Two A chromosome ring bivalents accompanied by a single B chromosome (arrow) at diplotene. Similar as on the A chromosomes HEI10 threads are evident on the self-paired B chromosome. Bar = 2 μm. **(C)** Typical twisted SC structures marked by ASY1, ZYP1, and HEI10 on an A bivalent at diplotene. **(C**_**1**_**)** The enlarged view of **(C)** shows the co-localization of the three proteins at the fragmented SC and indicates the ongoing desynapsis. Bar = 2 μm. **(C**_**2**_**,C**_**3**_**)** Besides weak HEI10 foci along ZYP1, two pronounced HEI10 spots are visible at higher magnification. The localization of such foci toward the bivalent connection sites at late diakinesis **(A**_**4**_**,D)** suggests the staining of crossovers. Note, the HEI10 spot in **(C**_**2**_**)** is localized slightly apart from the central element of the SC marked by ZYP1. Bar = 2 μm. **(D,D**_**1**_**)** Distinct HEI10 spots (arrowheads) on an A chromosome ring bivalent at late diplotene. Both spots are not located on SC residues and likely correspond to the HEI10 signals exclusively evident at late diakinesis **(A**_**4**_**)**. Bar = 2 μm. **(E)** Three A bivalents at late diakinesis show two HEI10 foci each. Instead, the single B chromosome (arrow) does not contain any HEI10 spots. Bar = 2 μm.

**Figure 7:**
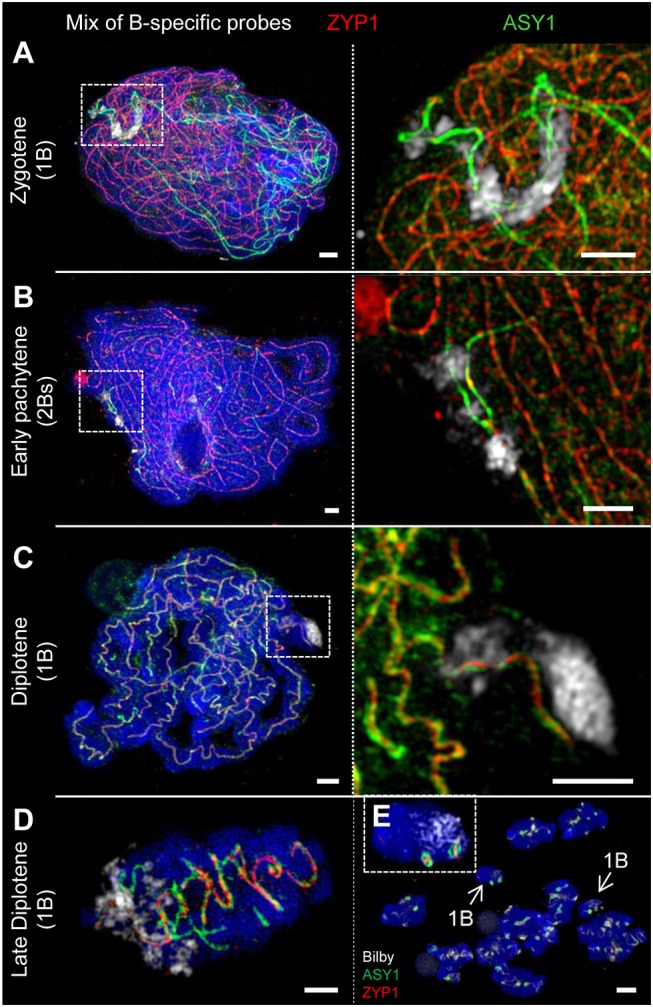
The SC structure of B chromosomes does not differ from that of As. Bs were detected by means of a B-specific FISH probe mix. The global chromatin was counterstained with DAPI. **(A)** At zygotene, ASY1 is associated with the B chromosome axis similar to that on As. The loading of ASY1 is independent of the B chromosome number, thus also occurring at single Bs performing intrachromosomal synapsis. Bars = 2 μm. **(B)** In case of 2 Bs, normal synapsis of both occurs. The enlargement shows a not yet synapsed B chromosome region indicated by two separate ASY1 strands with brighter fluorescence at early pachytene. Bars = 5 μm. **(C)** In absence of a homologous pairing partner, intrachromosomal synapsis of single B chromosomes takes place. The ASY1 and ZYP1 staining is identical to that of A bivalents during diplotene. Bars = 2 μm. **(D)** Similar to that of A bivalents at late diplotene (Figure 3A_4_) B bivalents show the retraction of ASY1 strands from the SC during desynapsis. Bar = 2 μm. **(E)** Two meiocytes (mixed by squashing) of rye carrying 1B chromosome each at diakinesis. Both Bs (arrows) can be distinguished from As by smaller size. The inset shows one of the B chromosomes with two typical ball-like residual structures of the SC complex. Bar = 5 μm.

**Figure 8:**
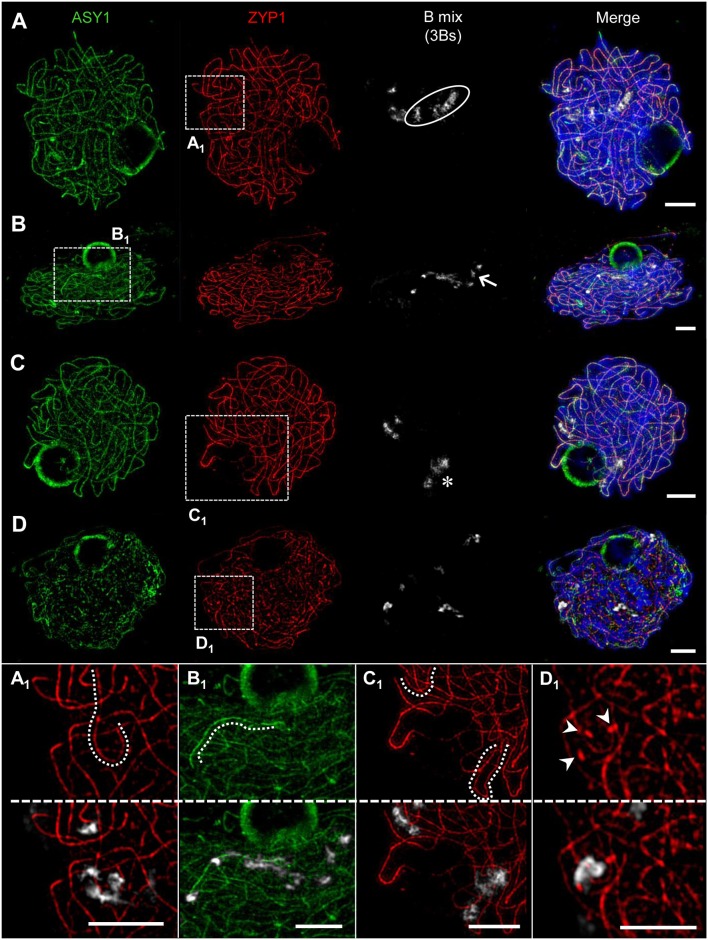
Pairing configurations of B chromosomes at pachytene. 3Bs were detected by means of a B-specific FISH probe mix, SCs with ASY1- and ZYP1-specific antibodies. Three different types of B chromosome arrangement were observed. Chromatin was counterstained with DAPI. **(A**) The Bs form one bivalent and one univalent. The bivalent **(A_**1**_)** shows normal synapsis, whereas the univalent B (circle) did not show any labeling by ASY1 or ZYP1 indicating the absence of a SC. **(B)** One B univalent and one B bivalent associate. While the bivalent (arrow) performs normal synapsis indicated by ZYP1, the univalent **(B**_**1**_**)** was exclusively loaded with ASY1. **(C)** Formation of one B bivalent and one B univalent. The bivalent (asterisk) performs normal synapsis, while the univalent B shows intrachromosomal synapsis revealed by ZYP1 labeling **(C**_**1**_**)**. **(D)** Three B univalents show a fragmented SC formation [**(D**_**1**_**)**, arrow heads]. Bars = 5 μm.

Compared to widefield microscopy, electron and super-resolution microscopy provide a significantly increased resolution, thus offering the analysis of plant chromatin and protein structures at the nanoscopic level (Baroux and Schubert, 2018). Here we used scanning electron microscopy (SEM; Figure 1) to obtain new insights in the structure of paired homologous chromosomes in prophase I meiocytes of rye. Although electron microscopy allows visualizing cell structures at a resolution of 1–2 nm it is challenging to label and localize DNA and proteins specifically (Baroux and Schubert, 2018). Therefore, we additionally applied fluorescence-based 3D-SIM to investigate chromatin and protein substructures in more detail (Figures 2–8). Compared to widefield microscopy a clearly increased resolution and the removal of out-of-focus blur has been achieved by SIM (Supplementary Figure 1). The localization and dynamics of the specifically stained SC components ASY1 and ZYP1, as well as the associated proteins NSE4A and HEI10 were monitored during prophase I at rye A and B chromosomes (Figures 3–8; Supplementary Figure 2; Supplementary Movies 2–4).

To identify centromeres and to conclude on the orientation of uni- and bivalents the A and B centromeres were labeled by the centromere-specific FISH probe Bilby (Francki, 2001) and CENH3 antibodies (Figures 2, 4B, 5B,E; Supplementary Figure 2; Supplementary Movies 1, 2).

### SEM Identifies the Organization of Synapsed Homologs

Cross-sections of meiocytes were analyzed by SEM. Similar to what was observed earlier on somatic barley metaphase chromosomes (Zoller et al., 2004a,b; Wanner et al., 2005) several chromatin clusters (chromomeres) were identified at the surface of the synapsed rye homologs (Figure 1A). During zygotene-pachytene the paired homologs are connected via a structure presumably representing the SC. Similar to lily, maize and human (Holm, 1977; Scherthan et al., 1998; Franklin et al., 1999) the SC of rye is located laterally to the chromatin of both homologs (Figure 1B).

### ASY1 and ZYP1 Form Typical Structures During SC Assembly and Disassembly

The dynamics of the synaptonemal complex during prophase I was monitored by immunolocalization of ASY1 and ZYP1 (Figures 3, 5A,D, 6–8; Supplementary Figure 1; Supplementary Movies 2–4). At zygotene, synapsis is initiated at several sites along both homologs. During the SC assembly, ASY1 is partially released from synapsed chromosomes resulting in substantially lower fluorescence intensity and diffuse ASY1 signals in the nucleoplasm at pachytene. Notably, apart from linear tracts disperse ZYP1 signals can also be detected, likely indicating yet unassembled proteins (Figure 3A_1_). At the beginning of pachytene synapsis completes and the SC tripartite structure is clearly visible (Figure 3A_3_). ASY1 signals appear as discontinuous stretches and spots with varying intensities. At late pachytene the ongoing chromatin condensation is accompanied by SC coiling, showing the most compact twisted structure at diplotene. The compaction of chromosomes also results in a more contiguous staining of ASY1. The first initiation of SC disassembly can be detected at late pachytene by the re-organization of ASY1 at single SC sites to form transient loop-like structures. At positions where both ASY1 strands dissociate from the SC, ZYP1 signals are no longer detectable indicating the local release of synapsis (Figures 3A_2−4_, 5A,D). During progression of SC disassembly at diplotene, ASY1 undergoes partial degradation resulting in fragmented ASY1 threads (Figures 3A_4_, 5D, 6A3,C; Supplementary Movie 4). At early diakinesis the SC fragments continue condensing, at which ASY1 winds up around residual ZYP1 fragments. Further shortening of these fragmented SCs progresses until 2–3 compact ball-like structures per bivalent remain at late diakinesis among the centromeres and at potential recombination sites (Figure 3, Supplementary Movie 2). The SC structures marked by ASY1 and ZYP1 disappear completely at the end of diakinesis (Figures 6A_4_,E).

In summary, we conclude that the SC structures composed by ASY1 and ZYP1 are involved not only in the establishment of synapsis. Obviously, they are also required to organize and stabilize the paired homologs during chromatin condensation until prophase I terminates.

### The SMC5/6 Complex δ-kleisin NSE4 Colocalizes to ZYP1 Within the SC During Synapsis

The SMC5/6 complex has been implicated to have versatile functions in meiotic processes, i.e. in recombination as well as in SC assembly and stability (Verver et al., 2016). To investigate the role of SMC5/6 complex subunits during prophase I we analyzed the distribution and dynamics of δ-kleisin NSE4. To confirm the specificity of the *A. thaliana* NSE4A antibodies (Zelkowski et al., 2019) and to exclude unspecific signal detection which could be induced by fluorescence crosstalk of ZYP1, we labeled rye meiocytes with NSE4A antibodies only. The detected twisted NSE4A labeling corresponds to the typical SC labeling visible at synapsed homologs during diplotene. Thereby, a color crosstalk caused by ZYP1 immunolabelling can be excluded (Figures 4B, 5B; Supplementary Figure 2). Concurrently, chromosomes were labeled by the centromere-specific FISH probe Bilby to identify centromeres, and the orientation of paired homologs (Figures 4B, 5B,E, 7; Supplementary Figure 2; Supplementary Movies 1, 2).

The simultaneous labeling of NSE4A and ZYP1 revealed a strong co-localization of both proteins at the central region of the SC (Figure 4A). At zygotene, NSE4A is present only along the synapsed homologs. When chromatin condensation progresses, the twisted structure of NSE4A follows the SC structure typically seen at diplotene. During SC disassembly at early diakinesis, NSE4A signals match to the spatial distribution of ZYP1 and can be detected exclusively on the remaining ZYP1-positive SC fragments (Figure 4A_1_). At late diakinesis typical co-localized ball-like structures of NSE4A and ZYP1 are evident (Figures 4A_2_,A_3_, 5C).

Our findings suggest that NSE4, together with ZYP1, is involved in the organization and stabilization of synapsis during prophase I in rye.

### The SC Is a Protein Structure Embedded in Chromatin

The immunolozalisation of ASY1, ZYP1, and NSE4A at the SCs and the absence of DNA-specific DAPI staining indicate that the inner SC is mainly a chromatin-free protein structure during prophase I (Figures 5A–C). This structure becomes visible at zygotene (Figure 3), and is present until late diakinesis (Figure 5C).

The resolution achieved by SIM allows measuring the width of the ASY1 and ZYP1 structures at different prophase I stages (Figures 5D,E). At diplotene the width of single ASY1 loops is approximately half of that of synapsed regions. This reflects the retraction of individual chromosome axes regions during the SC disintegration at diplotene. At diakinesis, ASY1 signal measurements indicate that the ball-like structures are established by the accumulation of separate ASY1 threads around a ZYP1 core (Figure 5E).

### HEI10 Localizes to the SC During Synapsis and Indicates Likely the Location of Recombination Sites at Late Diakinesis

Recently it has been shown, that the ZMM protein family member HEI10 is involved in homologous recombination, and that it marks class I crossover loci in a number of organisms such as rice, *Arabidopsis* and mouse (Ward et al., 2007; Chelysheva et al., 2012; Wang et al., 2012; Qiao et al., 2014). To examine whether HEI10 exercises the same function in rye, we labeled different stages of prophase I with ASY1, ZYP1, and HEI10 antibodies simultaneously (Figure 6).

At zygotene, distinct HEI10 foci were detected exclusively at the central region of the SC marked by ZYP1 (Figure 6A_1_). When synapsis is completed at pachytene, single HEI10 foci become more prominent and clearly distinguishable (Figure 6A_2_). At diplotene, when the progression of the SC disassembly results in SC fragmentation, HEI10 can be detected as numerous low-intensity foci located along the central element of the SC. Additionally, a few prominent foci slightly apart from ZYP1 were present. The localization of such foci toward the bivalent termini suggests a staining of potential crossover sites (Figures 6C,D; Supplementary Movie 4). At late diakinesis, ASY1 and ZYP1 signals disappear completely, but distinct HEI10 puncta remain at potential crossover loci. The quantification of HEI10 signals in 50 meiocytes at this stage resulted in a mean of 13.1 signals per cell (*SD* = 1.57). This value reflects the expected number of chiasmata observed in diploid rye by Jones (1967) and strongly suggests a detection of crossover sites by anti-HEI10.

### Active Centromeres and the SC Structure of Rye A and B Chromosomes Do Not Differ

FISH using the centromere-specific probe Bilby showed, as previously described by Banaei-Moghaddam et al. (2012), that the meiotic B centromeres exhibit an extended and diffuse Bilby signal distribution compared to those of A chromosome centromeres. Taking the signal size of an antibody recognizing the centromere-specific histone H3 variant CENH3 as a means to determine centromere activity (Wang and Dawe, 2018), the simultaneous labeling of meiocytes by Bilby and anti-CENH3 revealed that the actual size of active centromeres is similar between A and B chromosomes (Figure 2).

Rye Bs may occur in even or odd numbers ranging from 1 to 8 (Jones and Rees, 1982). Analysis of the SC structure revealed that ASY1 becomes loaded onto the B chromosome axis at early prophase I irrespective of the presence of a homologous partner (Figures 7A, 8). In case of 2Bs, a normal SC assembly accompanied by the incorporation of ZYP1 occurs at pachytene. However, the SC formation of Bs present in odd numbers may be impaired. Beside the absence of ZYP1 and/or ASY1 loading (Figures 8A_1_,B_1_), the intrachromosomal SC formation ranging from small clusters (Figure 8D_1_) to long SC stretches (Figures 7C, 8C_1_) was observed on univalent Bs. When prophase I progresses, B chromosome SCs show the same twisted structure evident on As (Figures 4B_2_, 7C,D). No differences between inter- and intrachromosomal SCs were observed. At diplotene, the SC disintegration, indicated by the retraction of ASY1, results in transient ASY1 threads, SC fragmentation and the subsequent formation of residual ball-like SC structures (Figures 4A_1−2_, 7D,E). The immunolocalization of NSE4A and HEI10 on Bs showed the co-localization of both proteins with ZYP1, and followed the above-mentioned SC dynamics (Figures 4, 6; Supplementary Movie 4). Nevertheless, HEI10 disappeared completely at the end of diakinesis (Figure 6E) and was absent on univalent Bs.

In general, we conclude that Bs form similar SCs as As. In addition, the SC formation of Bs may be impaired depending on the B chromosome number per meiocyte.

### Prophase I Pairing Configurations of B Chromosomes Depend on Their Number

The quantification of the meiotic pairing within PMCs of rye plants containing different Bs allowed revealing various types of B chromosome behavior. In case of one B chromosome, ASY1/ZYP1-positive SC fragments reflecting self-synapsis were detected on all univalents examined (*n* = 10 meiocytes). Plants with 2Bs (*n* = 12 meiocytes) showed regular SC assembly and bivalent formation. Only in one case two univalents were formed in such plants and fragmented SCs were observed on those univalents. Plants carrying 3Bs (*n* = 94 meiocytes) revealed three modes of SC formation during prophase I. Namely, 84% of their meiocytes had one bivalent and one univalent (Figures 8A–C), in 12.8% a clusters of all 3Bs was formed, and only 3.2% of the cells contained three univalents (Figure 8D). In case of plants carrying 4Bs (*n* = 121 meiocytes) the following configurations were observed: 64.5% of meiocytes contained only bivalents, in 29.7% multivalents joining up all Bs were formed, and 5.8% had one bivalent plus two univalents.

Altogether, the data indicate the influence of the B chromosome number on the pairing configurations of Bs.

## Discussion

### Synapsed Homologs Form Chromomeres and a Chromatin-Free SC

SEM has been proven to be a suitable tool to investigate the architecture of plant chromosomes at the nanoscopic level (Wanner et al., 1991; Iwano et al., 2003; Wanner and Schroeder-Reiter, 2008). Studies on somatic plant metaphase chromosomes based on protein and DNA staining followed by SEM allowed to establish the so-called “dynamic matrix model” (Wanner and Formanek, 2000; Wanner et al., 2005). The model proposes that the chromosomes are mainly composed of DNA packed in chromomeres (coiled solenoides) around a dynamic matrix formed by parallel protein fibers. This protein matrix may also contribute to form the chromatin-free axes/SCs during synapsis. The model was also shown to be applicable to meiotic chromatin of rye (Zoller et al., 2004a,b). In line with these reports, we observed tightly packed chromomeres at the surface of chromosomes from zygotene to diplotene in rye. Furthermore, our finding that the SC is localized outside and not enclosed by chromatin, suggests a lateral co-orientation of the chromatid axes prior to SC formation. Such a chromatin configuration can facilitate an unhindered loading of the AE proteins and the SC assembly. As a consequence, this results in a mainly chromatin-free proteinaceous SC structure that was previously documented in other organisms such as lily and maize (Holm, 1977; Dawe et al., 1994). Due to the high degree of compactness of meiotic chromosomes, the detection of matrix fibers would require an additional application of enzymes, such as proteinase K to loosen the chromatin (Zoller et al., 2004b).

### The Dynamics of ASY1 and ZYP1 Indicate an Assembly and Disassembly of the SC During Prophase I

Previous studies demonstrated that antibodies raised against the two SC proteins ASY1 and ZYP1 of *A. thaliana* are suitable for the detection of orthologous proteins in other plant species, e.g., in barley and rye (Mikhailova et al., 2006; Phillips et al., 2008, 2010). Here, we utilized ASY1 and ZYP1 to investigate homologous pairing events during prophase I in rye utilizing SIM. Synapsis is initiated in rye at telomeres and interstitial sites as previously reported (Abirached-Darmency et al., 1983). When synapsis occurs, the ASY1 signal intensity decreases severely. It cannot be excluded, that this observation results from a decreased accessibility of the ASY1 antibodies to the epitopes as a consequence of the SC assembly and chromatin compaction (Golubovskaya et al., 2006). However, a weak ASY1 staining in the nucleoplasm is detectable at early pachytene suggesting that ASY1 is partially removed from the AEs/LEs during synapsis. Similar observations were reported for various species. In rice, maize and budding yeast the signal intensity for the orthologs PAIR2, ASY1, and HOP1, respectively, also significantly decrease during synapsis (Smith and Roeder, 1997; Nonomura et al., 2006). However, in contrast to these species, rye ASY1 is not removed from the axes during pachytene. It remains at the SC until its disintegration, comparable to the orthologous proteins of barley and *Arabidopsis* (Armstrong et al., 2002; Phillips et al., 2012). Previous studies, using rye synaptic mutants as experimental material, reported that ASY1 and ZYP1 pre-assemble. It was hypothesized that these double layer tracts could be formed in wild-type rye as well before synapsis and later could interact to form the SC (Mikhailova et al., 2006; Phillips et al., 2008). In our study, we did not observe such a pre-alignment of SC fragments in wild-type rye meiocytes carrying accessory B chromosomes, but ASY1 located exclusively to the AE/LE elements in As and Bs. At zygotene, ZYP1 was incorporated at the CR of the SC in a zipper-like manner. Moreover, a diffuse staining of yet unassembled ZYP1 within the nucleoplasm was found, which decreased when synapsis has finished. These deviating observations could be due to the different rye genotypes studied, as well as the specificity of the different antibodies used, i.e., anti-maize in this study vs. anti-*Arabidopsis* ASY1 and ZYP1 in the previous one. Different slide preparation techniques, especially the fixation in 4 vs. 2% paraformaldehyde, as well as the increased resolution and detection sensitivity achieved by SIM, which allows more precise observations compared to confocal laser scanning microscopy, could also be crucial. During the progression of prophase I, we observed remarkable structural chromatin changes. At the end of pachytene, the SC adopts a twisted structure, consistent with previous studies (Fedotova et al., 1989; Mikhailova et al., 2006; Simanovsky et al., 2014). This coiling was not the result of helical winding of the chromosomes, because the SC structures did not form symmetrical spirals. Instead, it was a result of contracting chromatin.

According to the “dynamic matrix model” (see above) coiled solenoids bind to interconnected matrix fibers. During condensation, the matrix fibers may act in an actin/myosin-like manner, whereby the parallel arrangement of the matrix favors shortening and thickening of the chromosomes. We propose that a similar mechanism could occur in meiotic chromosomes. But in contrast to mitosis, meiotic chromosomes need to condense and separate two paired homologs. Therefore, it is plausible, that the SC does not only provide the platform for recombination but it may also link both homologs to synchronize the condensation process. By tethering chromomeres of all chromatids to a common axis a random chromatin organization may be prohibited. Given that chromosomes condense during complete prophase I, a successive compaction of the chromomeres is reached. As a consequence of the sterical restrictions the tension along the paired homologs increases and thus, causes the bending of the SC at pachytene. At late pachytene/diplotene when the tension increases further, a local repulsion of single LE occurs, apparent by the retraction of ASY1 threads, which form transient loops. Similar dynamic structures of ASY1 were recently described in meiotic chromosomes of wheat and barley (Colas et al., 2017). During these processes ZYP1 disappears from the CR and disintegration of the SCs emerges. By disassembly of the SC, both homologs become separated piecewise. Possibly, the ball-like SC structures of ZYP1 and ASY1 are formed and remain to counteract the tension throughout diakinesis. Thus, a stabilization of the bivalents is achieved and the premature separation of recombination sites and centromeres may be prevented. Last traces of the SC are lost at the end of diakinesis, when chiasmata and centromere formation are fully accomplished.

The persistence of SC components at centromeres, additionally to the recombination sites during their disassembly in late prophase I, has also been described in budding yeast, *Drosophila*, mouse and human (Bisig et al., 2012; Qiao et al., 2012; Kurdzo and Dawson, 2015). Our finding, that SC components accumulate also at centromeres during the SC disassembly in plants indicates a conserved phenomenon, important to perform proper meiotic chromosome segregation.

In many organisms, including protists, fungi, animals, and plants the aggregation of SC-related material to form so-called polycomplexes was reported (Zickler and Kleckner, 1999). Because our study shows that the ball-like structures result directly from SC disassembly, we conclude that they are not SC-independent aggregations of SC-related proteins as present in polycomplexes (Zickler and Kleckner, 1999). Despite intense studies, polycomplexes have never been reported in prophase I stages of rye. Therefore, we exclude this sort of interpretation.

### The SMC5/6 Complex δ-kleisin NSE4 Seems to Be Required for Synapsis and Recombination

NSE4 is the crucial non-SMC δ-kleisin component of the SMC5/6 complex, and therefore can be considered as a reliable marker for its localization (Palecek et al., 2006). In *A. thaliana*, two orthologs, *Nse4A* and *Nse4B*, were identified. Both genes are expressed in different tissues and are required to realize complete fertility. However, *Nse4A* is the more essential gene (Watanabe et al., 2009; Zelkowski et al., 2019). Despite the increasing knowledge about SMC5/6 of non-plant eukaryotes (Verver et al., 2016), the immunohistochemical analysis of the NSE4 distribution and its dynamics during meiosis in plants was challenging so far due to the lack of specific antibodies. In the present study, we localized for the first time the SMC5/6 complex subunit NSE4 in prophase I of rye, using antibodies raised against NSE4A of *A. thaliana* (Zelkowski et al., 2019). The detection of NSE4 from early zygotene until late diakinesis is similar to the localization pattern found in mammalian meiosis (Verver et al., 2016). In rye, NSE4 co-localizes with the TF protein ZYP1, indicating the restriction of NSE4 to synapsed chromosomes. Consistent observations were described in mice, where a SYCP1-dependent loading of SMC6 occurs (Gomez et al., 2013), and in human, where SMC5/6 localizes to synapsed chromosome axes (Verver et al., 2014). By its recruitment to synapsed axes, the SMC5/6 complexes might facilitate the formation and/or stabilization of synapsis in rye. Moreover, in *C. elegans*, fission and budding yeasts the SMC5/6 complexes are involved in homologous recombination and proper chromosome segregation (Pebernard et al., 2004; Bickel et al., 2010; Wehrkamp-Richter et al., 2012; Copsey et al., 2013; Lilienthal et al., 2013; Xaver et al., 2013). Our observed localization pattern of NSE4, especially at the late ball-like SC structures during diakinesis, might also indicate a function of SMC5/6 complexes in homologous recombination of rye.

The involvement in synapsis has also been proven for the meiotic α-kleisin of the SMC family complex cohesin. Its presence in prophase I was shown in plants such as *Arabidopsis* (Cai et al., 2003), tomato (Qiao et al., 2011), rice (Zhang et al., 2006; Shao et al., 2011), and in addition its co-localization to ZYP1 was proven in *Luzula* (Ma et al., 2016). These findings support the importance of SMC complex proteins for proper meiosis.

### HEI10, a Marker for Class I Crossovers in Rye?

In mice, the two RING-family E3 ligases HEI10 and RNF212 were shown to be essential for recombination (Reynolds et al., 2013; Qiao et al., 2014). In contrast to mammals, plants possess only one member of the broad RNF212/HEI10 protein family (Toby et al., 2003; Ward et al., 2007; Chelysheva et al., 2012; Wang et al., 2012; Rao et al., 2017). During meiosis of *A. thaliana* and rice, HEI10 proteins label the sites of class I crossovers (Chelysheva et al., 2012; Wang et al., 2012; Ziolkowski et al., 2017).

Our study provides evidence that antibodies raised against HEI10 of *O. sativa* (Wang et al., 2012) detect the corresponding proteins in rye. In rice, a punctate pattern of HEI10 occurs in early leptotene. Importantly, a linear distribution of signals alongside of ZEP1 (ZYP1 ortholog of rice) can be observed during synapsis, but disappears at diplotene. From late pachytene to diakinesis, additional prominent HEI10 foci were localized at the chromosomes, presumably marking class I crossover sites (Wang et al., 2012). Similar results were obtained in *A. thaliana* (Chelysheva et al., 2012). The localization of the orthologous rye protein found in our study differs from those reports, as HEI10 foci were not detectable before the onset of ZYP1 installation/loading. Thus, a crucial role of HEI10 at pre-synaptic events of recombination seems to be unlikely in rye.

Recent studies in *Sordaria* and mice revealed a similar localization pattern of HEI10 as seen in rye, by being associated only with SCs, although HEI10 is not a SC component. In both species, it was shown that HEI10 becomes engaged after the Mer3/MLH- and/or DMC1-mediated homolog pairing, and seems to regulate post-synapsis steps of meiotic recombination via a SUMO-ubiquitin switch (Storlazzi et al., 2010; De Muyt et al., 2014; Qiao et al., 2014). In *Sordaria*, HEI10/MSH4 foci were classified by morphology and dynamics, and proposed to mark three different types of recombination complexes: early and late SC-associated nodules, as well as non-nodule associated interactions (De Muyt et al., 2014). Previous TEM studies characterized two morphologically different types of recombination nodules: one at early and the second at late pachytene of rye (Abirached-Darmency et al., 1983). Given the lower resolution of SIM, the differentiation of these nodule types during pachytene of rye is not feasible. Nevertheless, it is tempting to assume that our observation of high fluorescence intensity HEI10 foci from pachytene on correspond to such recombination nodules, whereas the weak foci represent axis-associated HEI10 involved in the SUMO-ubiquitin switch. This assumption is supported by the distinct HEI10 foci present at late prophase I. During SC disassembly at diplotene, both types of HEI10 foci are clearly distinguishable, i.e. week signals associated with the remaining SC fragments, as well as prominent foci located toward the end of the bivalents either on or in close proximity to the SC. HEI10 foci apart from the SC may indicate recombination sites no longer connected to the SC due the proceeding chromosome condensation. After the complete disintegration of the SC at the end of diakinesis, only prominent HEI10 foci still persist. The mean number of those foci (13.1; *SD* = 1.57; *n* = 50 cells) matches the observed number of chiasmata in rye (Jones, 1967; Naranjo and Lacadena, 1980).

In short, we assume that HEI10 may be the first recombination marker identified in rye, most likely labeling class I crossovers.

### A Model for the Behavior of Rye Chromosomes During Prophase I

Despite extensive studies on the mechanisms of SC establishment and disassembly in various organisms, it is still challenging to decipher the complex events underlying the SC formation and recombination.

**Figure 9:**
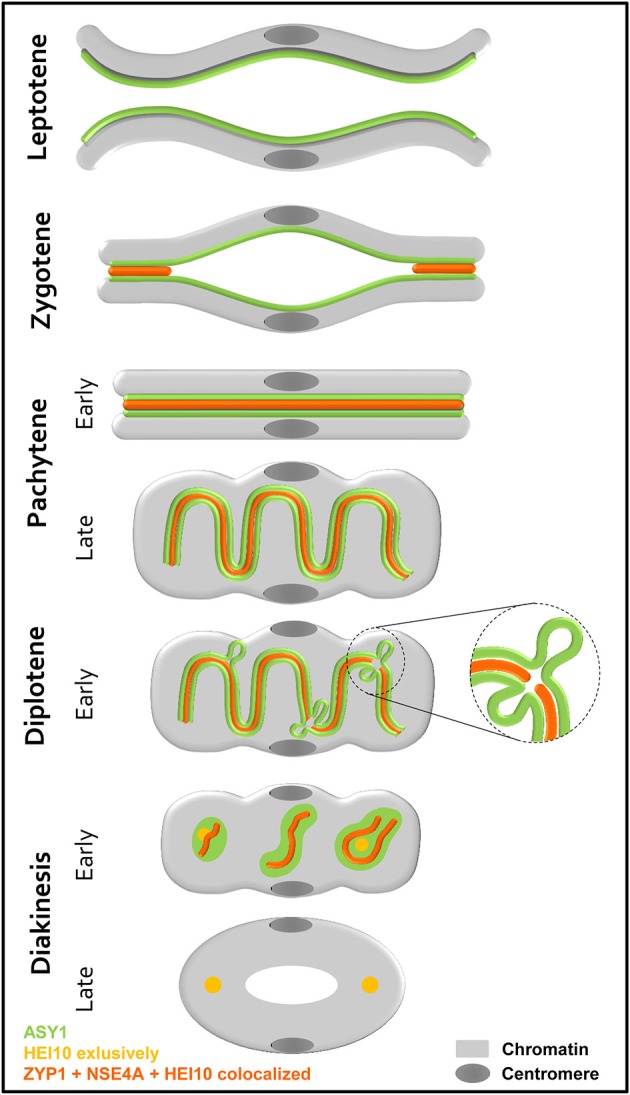
Scheme showing the behavior of two homologous chromosomes, together with the localization of the SC proteins ASY1, ZYP1, NSE4A, and HEI10 during prophase I. Before synapsis, ASY1 is loaded along each chromosome axis at leptotene. During zygotene, the assembly of the SC occurs at multiple sites of the homologs. Thereby, ZYP1, NSE4A, and HEI10 are incorporated at the central region. Synapsis completes at the beginning of pachytene visible by the tripartite structure consisting of two lateral elements enclosing the central region. The ongoing degree of condensation observed throughout prophase I, is accompanied by a coiling of the SC at late pachytene. At diplotene, the onset of SC disintegration is accompanied by the retraction of ASY1 forming transient loops. At positions, where both ASY1 threads are retracted from the SC, ZYP1, NSE4A, and HEI10 disappear (enlarged). The progression of the SC degradation results in fragmented ball-like SC structures at early diakinesis. In addition to the ZYP1 and NSE4A labeling at the crossover sites and in between the centromeres, distinct HEI10 foci marking crossover sites are evident. At late diakinesis, the SC disassembly is completed, and ASY1, ZYP1, and NSE4A disappeared completely. Only the sites of crossovers remain clearly marked by HEI10.

Based on our findings, we propose a model for the homologous chromosome behavior during prophase I based on the observed dynamics of ASY1, ZYP1, NSE4A, and HEI10 (Figure 9). At leptotene, the AE/LE-associated protein ASY1 is loaded onto the chromosome axes prior to SC formation. As synapsis occurs, ZYP1, NSE4A, and HEI10 become incorporated at the central region of the SC. Further, the ongoing condensation of the chromatin throughout prophase I leads to the coiling of the SC at late pachytene. The following disintegration of the SC is accompanied by the retraction of ASY1 from the central region, most likely as a result of the increasing tension on chromatin caused by condensation. At positions where both ASY1 threads are retracted, the CR is dissolved and ZYP1, NSE4A, and HEI10 are no longer detectable (Figure 9, enlarged). As a consequence, SC fragmentation and the formation of ball-like structures can be observed at early diakinesis. Additionally to weak HEI10 signals on the remaining SC, distinct foci at both ends of the bivalent reflecting the sites of crossover are detectable. Colocalizing ASY1, ZYP1, and NSE4A proteins are also present in between the homologous centromeres. But here, HEI10 is missing indicating the absence of crossovers. At late diakinesis, the SC disassembly is completed as indicated by the disappearance of ASY1, ZYP1, and NSE4A at the crossover sites as well as at the centromeres. Only the class I crossovers remain marked by HEI10.

### During Prophase I Bs Behave Like A Chromosomes

For decades the origin of rye B chromosomes remained enigmatic. The application of next generation sequencing revealed that Bs originate from several A chromosome fragments and an accumulation of various repeats and insertions of organellar DNA (Martis et al., 2012). Moreover it was shown, that Bs possess their own evolutionary pathways and that they accumulate high copy sequences, allowing to identify rye Bs during prophase I by FISH (Klemme et al., 2013). By combination of antibodies directed against ASY1, ZYP1, NSE4A and HEI10 with B-specific FISH probes, we analyzed the SC composition of Bs. Previous EM studies of the SC formation in various species revealed substantial differences between the meiotic pairing of As and Bs dependent on their number (Jenkins, 1985; Switonski et al., 1987; Kolomiets et al., 1988; Shi et al., 1988; Santos et al., 1993). Moreover, studies of the Chinese racoon dog demonstrated diverging SC structures of As and Bs. Namely during pachytene the SC axes of the B chromosomes are significantly denser than those of the As. Depending on the number of Bs, bivalents and multivalents could be formed. If three Bs were present in parallel, the alignment of all three SC axes might occur (Shi et al., 1988). For rye Bs it was shown, that in contrast to 2Bs, univalents and higher B chromosome numbers form either intrachromosomal SCs, or perform the segmental pairing in multivalents. In contrast to the Chinese racoon dog, SCs of Bs formed by more than two AEs/LEs have never been observed (Santos et al., 1993). In rye with increasing B numbers altered SC formation occurs unrelated to the mean number of A-located chiasmata (Diez et al., 1993), while in *Crepis capillaris* SC irregularities of As correlate with defective A chromosome pairing when 4 Bs are present (Jones et al., 1991). Our study revealed that in general the SC composition of Bs does not differ from that of As, as proven on meiocytes containing 2Bs. All four proteins investigated localize to the SCs of Bs and manifest the same dynamics as described for As. Despite their different nature compared to As, rye Bs utilize obviously the same protein structures to ensure meiotic pairing and proper chromosome condensation. Rye plants comprising less or more than 2Bs show also a similar SC composition independent of segmental or intrachromosomal pairing. Obviously, by producing the same SC structures the Bs try to fulfill the pairing requirements. Similar observations were described also for univalent A chromosomes, e.g., in barley, lily, wheat, and maize (Gillies, 1974, 1981; Holm, 1977; Hobolth, 1981). Only in very rare cases, the formation of SCs fails and either ASY1 only or none of the proteins were detectable. Previous studies suggested that the intrachromosomal pairing is a non-homologous process and has no genetic consequences due to the lack of recombination (Santos et al., 1993).

The prophase I meiotic pairing configurations of rye Bs were found to be genotype-dependent and are linked to the efficiency of B chromosome transmission to the next generation. Whereas bivalent formation secures the successful transmission of Bs, uni-, and multivalents have a much lower transmission rate (Jiménez et al., 2000). The rye variety “Paldang” we analyzed has a B transmission rate of about 20% (Romera et al., 1989).

In summary, we conclude that despite the deviating chromatin composition A and B chromosomes establish similar SC structures to perform pairing in prophase I.

## Data Availability

All datasets for this study are included in the manuscript and the Supplementary Files.

## Author Contributions

SH, AH, VS, and EM conceived the study and designed the experiments. SH, VS, MZ, EM, and CK performed the experiments. SH and VS wrote the manuscript. All authors read and approved the final manuscript.

### Conflict of Interest Statement

The authors declare that the research was conducted in the absence of any commercial or financial relationships that could be construed as a potential conflict of interest.

## Supplementary Material

Supplementary Figure 1 SIM improves the resolution, and thus the identification of SC nanostructures significantly. Chromatin was stained with DAPI. **(A)** Comparison of ASY1 and ZYP1 immunosignals at zygotene acquired by conventional widefield microscopy and SIM. The increased resolution of SIM reveals more nanostructures and improves the co-localization analysis by higher precision. Bar = 2 μm. **(A**_**1**_**)** Enlarged region showing clearly interstitial synapsis (arrows) by SIM. Bar = 1 μm. **(B)** Widefield imaging of the ball-like ASY1 and ZYP1 structures at late diakinesis. Bar = 5 μm. **(B**_**1**_**)** SIM delivers a clearly increased substructural information compared to widefield microscopy. ZYP1 is embedded into a ball of ASY1. Bar = 0.5 μm.

Supplementary Figure 2 The exclusive NSE4A antibody application to meiocytes proves that the detected signals are not a result of fluorescence crosstalk. NSE4A co-localizing to ZYP1 (Figure 4) shows without a ZYP1 labeling the typical twisted NSE4A signals during diplotene, thus excluding a fluorescent crosstalk possible via double labeling. (Peri)centromeric regions of the homologs are marked by Bilby FISH probes. Chromatin was stained with DAPI. Bar = 5 μm.

Supplementary Movie 1 Meiocyte at zygotene containing seven A and two B bivalents labeled with the centromere-specific markers Bilby (green) and CENH3 (red). The B bivalents can be identified by their increased Bilby dispersion (below). See also Figure 2.

Supplementary Movie 2 At diakinesis ZYP1 threads (red) are embedded in three ball-like ASY1 (green) structures possibly stabilizing the centromeric region (white, marked by Bilby) and the two recombination sites at both chromosome arms (see also Figure 5E). The movie shows a single bivalent.

Supplementary Movie 3 Image stack of a meiocyte showing the distribution of ASY1 (green) and ZYP1 (red) at the SCs during zygotene (see also Figure 4A).

Supplementary Movie 4 ASY1 (green), ZYP1 (red), and HEI10 (white) colocalize at the SCs of two A bivalents and a smaller self-paired B chromosome (below) at diplotene (see also Figure 6B).
