# Supplementary figures and images for: Ultrastructure and Dynamics of Synaptonemal Complex Components During Meiotic Pairing and Synapsis of Standard (A) and Accessory (B) Rye Chromosomes
Source: Front Plant Sci. 2019 Jun 20;10:773. doi: 10.3389/fpls.2019.00773 (PMC6596450; doi:10.3389/fpls.2019.00773)

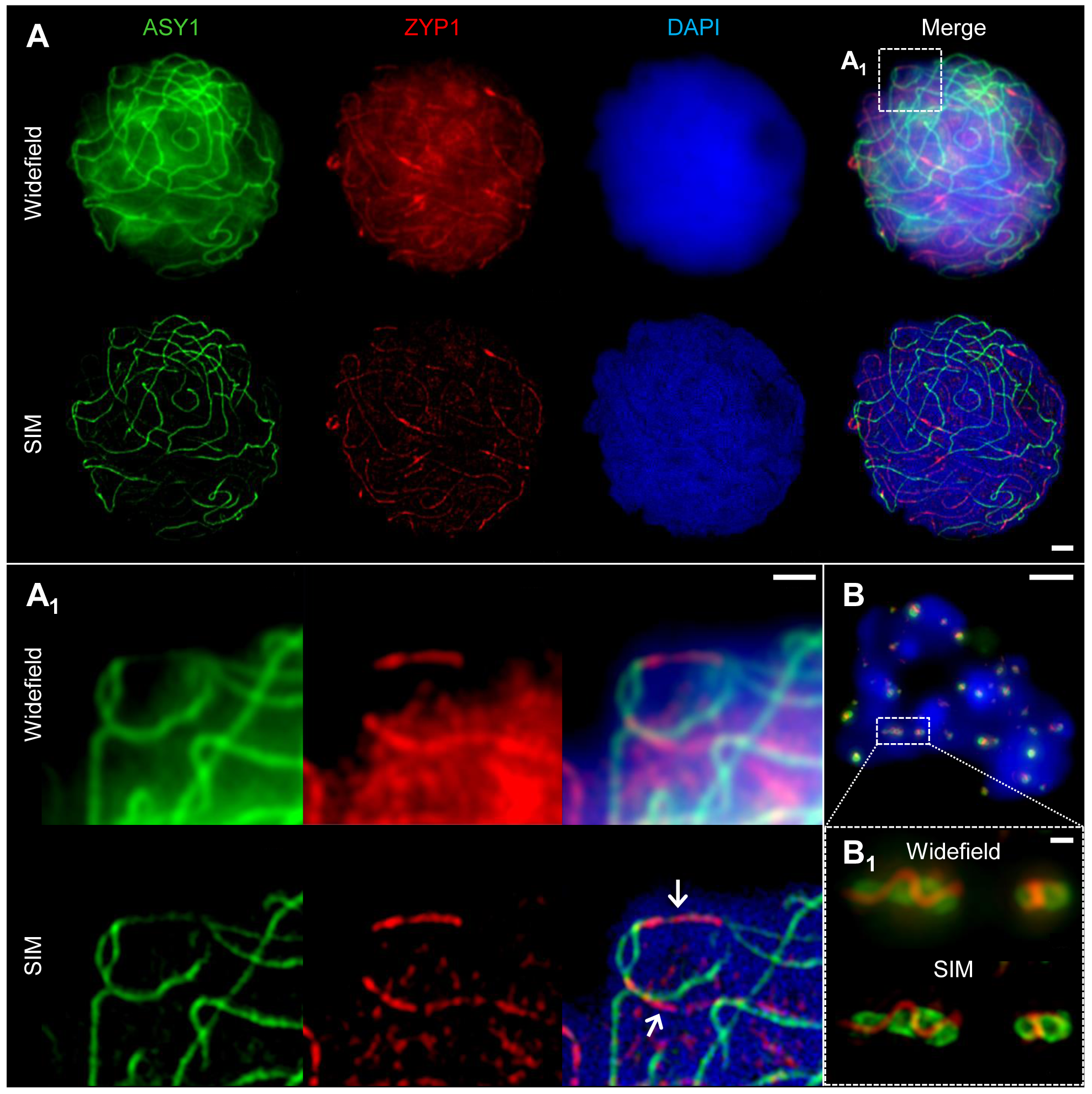

Supplement: Supplementary Figure 1 — SIM improves the resolution, and thus the identification of SC nanostructures significantly. Chromatin was stained with DAPI. (A) Comparison of ASY1 and ZYP1 immunosignals at zygotene acquired by conventional widefield microscopy and SIM. The increased resolution of SIM reveals more nanostructures and improves the co-localization analysis by higher precision. Bar = 2 μm. (A1) Enlarged region showing clearly interstitial synapsis (arrows) by SIM. Bar = 1 μm. (B) Widefield imaging of the ball-like ASY1 and ZYP1 structures at late diakinesis. Bar = 5 μm. (B1) SIM delivers a clearly increased substructural information compared to widefield microscopy. ZYP1 is embedded into a ball of ASY1. Bar = 0.5 μm. [file Image_1.TIF]

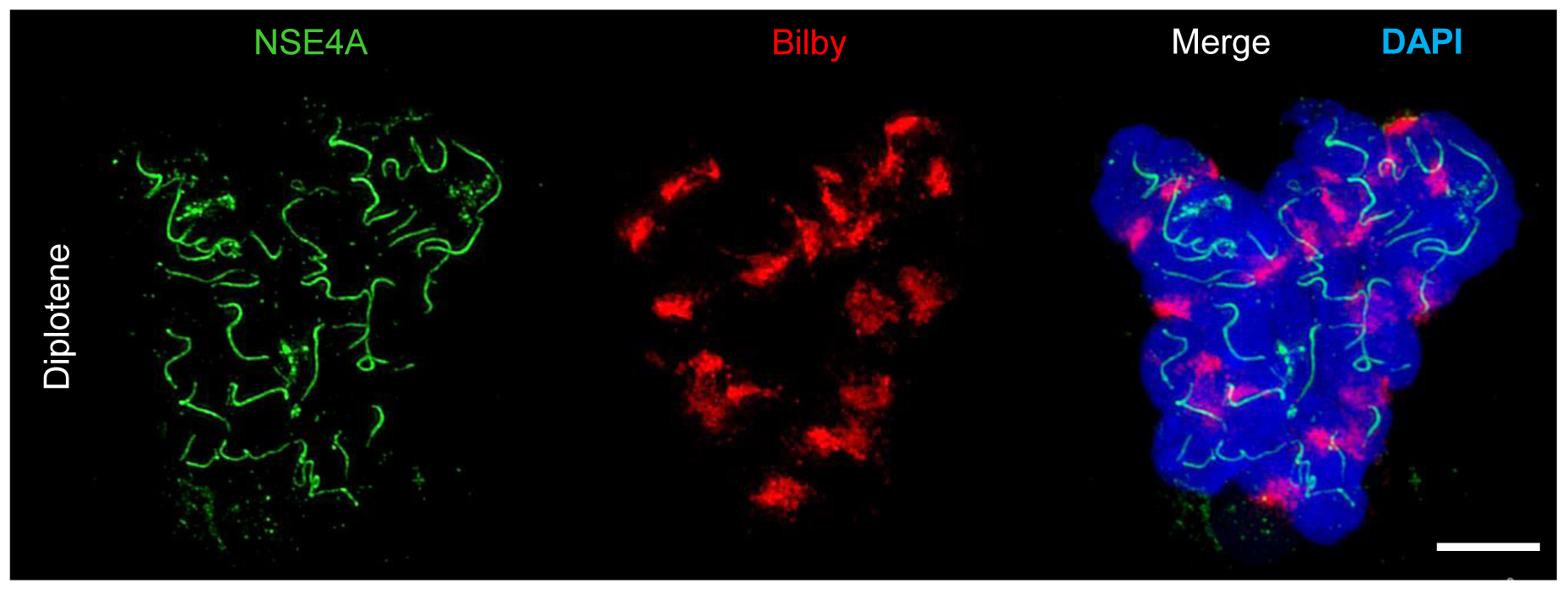

Supplement: Supplementary Figure 2 — The exclusive NSE4A antibody application to meiocytes proves that the detected signals are not a result of fluorescence crosstalk. NSE4A co-localizing to ZYP1 (Figure 4) shows without a ZYP1 labeling the typical twisted NSE4A signals during diplotene, thus excluding a fluorescent crosstalk possible via double labeling. (Peri)centromeric regions of the homologs are marked by Bilby FISH probes. Chromatin was stained with DAPI. Bar = 5 μm. [file Image_2.TIF]
